# Social Robots in Applied Settings: A Long-Term Study on Adaptive Robotic Tutors in Higher Education

**DOI:** 10.3389/frobt.2022.831633

**Published:** 2022-03-15

**Authors:** Melissa Donnermann, Philipp Schaper, Birgit Lugrin

**Affiliations:** Human-Computer Interaction, Institute of Computer Science, University of Wuerzburg, Wuerzburg, Germany

**Keywords:** human–robot interaction, adaptive tutoring, higher education, robot-supported training, technology-supported education, robotic tutor

## Abstract

Learning in higher education scenarios requires self-directed learning and the challenging task of self-motivation while individual support is rare. The integration of social robots to support learners has already shown promise to benefit the learning process in this area. In this paper, we focus on the applicability of an adaptive robotic tutor in a university setting. To this end, we conducted a long-term field study implementing an adaptive robotic tutor to support students with exam preparation over three sessions during one semester. In a mixed design, we compared the effect of an adaptive tutor to a control condition across all learning sessions. With the aim to benefit not only motivation but also academic success and the learning experience in general, we draw from research in adaptive tutoring, social robots in education, as well as our own prior work in this field. Our results show that opting in for the robotic tutoring is beneficial for students. We found significant subjective knowledge gain and increases in intrinsic motivation regarding the content of the course in general. Finally, participation resulted in a significantly better exam grade compared to students not participating. However, the extended adaptivity of the robotic tutor in the experimental condition did not seem to enhance learning, as we found no significant differences compared to a non-adaptive version of the robot.

## 1 Introduction

Self-directed and lifelong learning is the basis of today’s knowledge society. However, this is challenging for many learners, as it requires a high degree of motivation and attention. In adult education, the requirements for a proactive and self-directed learning style are particularly high, especially at universities. In the higher education context, the transfer of knowledge is often unidirectional and offers little room for individualization and interactivity. Especially for students in their first semesters, self-study is usually a big challenge. Effective learning strategies and self-motivation are required, but often have to be learned and refined over time. This challenge was even more prominent during the last year due to the COVID-19 pandemic, and an accompanying shift to online teaching, which further reduced individual support and feedback from teachers.

Several approaches to support learners in this situation are based on technology-enhanced education. However, most current systems have a significant weakness: the social aspect of learning is not sufficiently addressed. It has already been shown that social interaction between teacher and students has a positive influence on many aspects of learning, such as academic performance, intellectual ability, and especially motivation (Tiberius and Billson, 1991; Witt et al., 2004; Velez and Cano, 2008).

Motivation, which is often used as a synonym for intrinsic motivation, can be considered as a basic prerequisite for learning (van Roy and Zaman, 2017). Self-determination theory (Deci and Ryan, 2011) has been highly influential in learning contexts, indicating a beneficial effect of autonomy-supportive classrooms on intrinsic motivation. However, intrinsic motivation only increases gradually (Vallerand et al., 1992; Deci and Ryan, 2000), and only if certain circumstances are met (Tsigilis and Theodosiou, 2003). In detail, the needs for competence, relatedness, and autonomy have to be satisfied (Deci and Ryan, 2011). To achieve these circumstances for different learners, who might show inter-individual differences within their requirements for need satisfaction, adaptivity of the system is required. Social robots are a special kind of pedagogical agents that have the potential to support students in their learning process and to bring social aspects into the learning situation through their ability to show social behavior (Saerbeck et al., 2010; Belpaeme and Tanaka, 2021). Therefore, a social robot can combine the advantages of technology-supported education and some aspects of a physically present teacher as well as supplying its own specific advantages, such as causing learners to feel less ashamed when making mistakes (Donnermann et al., 2020). As such, social robots have the potential to enhance aspects of learning such as motivation, attention, and even fun (Van Minkelen et al., 2020).

Social robots are designed to interact with humans in a natural, interpersonal way (Breazeal et al., 2016) and to support their users through social interaction, often in educational contexts. Research suggests that the physical presence of a robot has a positive impact on learning outcomes relative to virtual representations or no learning support (Leyzberg et al., 2012; Kennedy et al., 2015; Li, 2015). In the context of learning, robots can take on two different roles: Either as a passive educational tool (e.g., using a robot to teach students programming of a robot) or as an active participant in the learning situation. As a participant, the robot can actively shape the learning process through social interaction. Acting as a learning assistant, the robot supports learners primarily by providing instructions as well as explaining and controlling the learning activity (Belpaeme et al., 2018; Mubin et al., 2013).

Research on social robots in education has shown promising results in terms of affective and cognitive benefits but has so far focused mainly on children (Belpaeme et al., 2018). However, recent research is increasingly looking at adults including the application of social robots in adult learning to benefit learners (e.g., Weber and Zeaiter, 2018; Donnermann et al., 2021a; Rosenberg-Kima et al., 2020; Velentza et al., 2021a). Due to their ability to show social behavior and personalize the learning experience, social robots bear great potential to address the challenges of self-directed learning in higher education and to increase motivation by addressing the basic psychological needs (Van Minkelen et al., 2020).

Pfeifer and Lugrin (2018) conducted a study in the field of adult education and created a robot-supported learning environment to teach HTML basics to university students. Results reveal that all participants improved their knowledge, and female participants, which learned (stereotypically male learning materials) with a female robot, benefited the most. In a different study, Deublein et al. (2018) set up a learning environment to teach Spanish as a second language to students with the support of a robotic tutor. Albeit there was no significant difference, results indicated that a robot had the tendency to benefit learning outcomes more if it tried to increase confidence and satisfaction. Similarly, Donnermann et al. (2021a) evaluated the effect of adding a supportive robotic tutor to a learning environment on motivation and engagement in a university context. However, ceiling effects in engagement or motivation, which were particularly high in both conditions, resulted in a lack of significant effects. This illustrates the challenge to demonstrate the effects of a robot tutor when implementing high-quality learning environments as control groups. A slightly different approach in university teaching was used by Rosenberg-Kima et al. (2020), who implemented a social robot to facilitate collaborative group activity of students, and compared it to a pen and paper, and a tablet condition. Qualitative data showed subjective benefits of the robot such as perceived objectivity, efficiency, and improved time management. Focusing more on the effect of a robot’s personality traits, its body movements and attitude, Velentza et al. (2021b) conducted an experiment with university students in which a Nao robot performed a storytelling exercise. Participants preferred the robot with a cheerful personality and expressive body movements for future collaboration. Velentza et al. (2021a) compared a robot to a human lecturer in the field of higher education. They found a higher level of knowledge acquisition when lectured by the human tutor but a higher level of enjoyment and facial surprise expressions when receiving a lecture from the robot tutor. After a second session with the robotic tutor, knowledge acquisition and level of enjoyment increased compared to students who learned with the robot for only one session. Lei and Rau (2021) investigated how university students deal with feedback from a robot. In a dance learning scenario, a NAO robot was used as an instructor to teach dance movements and provide performance feedback to learners. The results showed that participants accepted both positive and negative feedback from the robot tutor, while participants were more likely to accept positive feedback. Compared to the negative feedback, the robot tutor’s positive feedback had a greater influence on the learner and could improve the learner’s motivation, enthusiasm, encouragement, confidence, and happiness, which demonstrate the possible impact of a robot’s teaching style on students’ behavior.

Integrating social robots in adult education can be technically more challenging because adult learners have higher demands on the robot’s abilities (Belpaeme and Tanaka, 2021) to actually feel supported in the learning process of complex learning materials. Because learning is a very individual process, adaptivity of a tutoring system is important to meet the needs of each learner. Therefore, adaptive intelligent tutoring systems aim to adjust the system to the user with the aim to provide the same benefits as one-to-one instructions (Phobun and Vicheanpanya, 2010). Based on the learners’ performance and actions in the learning environment, instructions are personalized to the learners’ knowledge level, learning styles, or emotional state.

Adapting the robot’s behavior can have positive effects on the learning process and the perception of the robot (Leyzberg et al., 2014). Based on the learners’ performance, the robot can shape the interaction, can provide encouragement, or can empathize with the learners depending on their answers (Belpaeme and Tanaka, 2021). Leyzberg et al. (2014) set up an experiment in which participants solved logic puzzles with a personalized or non-personalized robot tutor while the solving time was tracked. Even simple personalized assistance by a robot tutor could help to solve tasks more quickly and personalization led to behavioral differences of the learner and thus improved the human–robot interaction. A further study by Leyzberg et al. (2018) supports the assumption that personalizing the robot’s behavior is beneficial as children who received personalized lessons by a robot tutor over five sessions outperformed children who received random lessons. Schodde et al. (2017) implemented an adaptive robot-supported language tutoring for adult learners. Results showed that the score of correct answers during the training was higher when learning with the adaptive robot. These findings promise great potential in the context of university teaching, especially if individual support by lecturers is limited.

In our previous research, we conducted two field studies on the use of robotic tutors in university teaching as a voluntary offer accompanying a course (Donnermann et al., 2020; Donnermann et al., 2021b). In a first experiment, the social robot acted as a tutor to help students with exam preparation to an ongoing course. Qualitative interviews showed that participants praised the offer and the students showed high interest for the robotic tutoring. This benefit was supported by quantitative data, as participants who took part in the robotic tutoring performed significantly better in the exam compared to students who did not participate. However, participants wished for more individualization (Donnermann et al., 2020).

To further improve the robotic tutoring, we conducted a second field study and implemented an adaptive version of the robotic tutor, comparing it to a non-adaptive version over one tutoring session. However, due to the COVID-19 pandemic, the tutoring session was set up as a live video call between the robot and the participant. To examine potential differences in motivational and attentional effects as well as performance in the exam, it was compared to a non-adaptive control condition. Overall, the results indicate subjective and objective benefits of the robotic tutoring such as a significantly better exam performance of the participants relative to the rest of the course. Furthermore, the subjective use of the adaptive robotic tutor was rated significantly higher than for the non-adaptive robot. We found no significant effects of the manipulation on the other measurements, but high ratings of the robotic tutoring in general. One weakness of the study was the online setting, as we do not expect the robot to develop its full potential in an online setting, and the adaptive elements, which were possibly too subtle to be sufficiently perceived (Donnermann et al., 2021b).

In the present study, we want to continue this successful approach of integrating a social robot in university teaching but address the shortcomings of this last study and improve the robotic tutor and evaluate it in an *in situ* setup. Therefore, we implemented additional and more pronounced adaptive elements and tested the adaptive social robot in a one-to-one presence tutoring over several sessions, spread over several months, to investigate long-term effects.

For a setting within higher education, it is also of interest to consider a long-term approach (Belpaeme and Tanaka, 2021) to allow integration into learning behavior rather than a sole intervention. Because the learning process in an applied setting extends after an experimental manipulation, changes over time should be considered in detail. In the field of social robotics, the line between a short-term interaction study and a long-term study is not clear. Leite et al. (2013) state that an interaction can be considered as “long-term” when the user becomes familiarized with the robot to a point that her perception of such robot is not biased by the novelty effect anymore, but define no certain number of sessions or time. However, there are arguments that after 1–2 sessions to 1–2 weeks, novelty has worn off as assessed by increased boredom [e.g., Salter et al. (2004)]. Therefore, we consider our study design with three sessions over a period of 10 weeks to also investigate long-term effects.

With our ongoing series of field studies over one semester, we want to contribute to a better understanding on how to apply and design social robots in the university context. To the best of our knowledge, this research approach is unique in adult education, not only because the study is conducted in a field setting in relation to university education, but also because it takes place over several sessions to overcome issues caused by novelty and can therefore also detect changes over time. By integrating an adaptive robotic tutor into the module across several learning sessions during a semester, we not only aim to investigate the effects of its adaptivity, but also focus in its applicability in general. Based on previous and related work, we propose the following hypotheses:

• H1: Participants’ performance in the exam at the end of the semester is better when learning with the adaptive robotic tutor compared to participants learning with a non-adaptive tutor and compared to the average course performance.
• H2: Participants perceive a higher subjective knowledge gain when learning with an adaptive robotic tutor than participants learning with a non-adaptive robotic tutor.
• H3: An adaptive robotic tutor supports participants’ motivation more than a non-adaptive robotic tutor.
• H4: An adaptive robotic tutor is rated better concerning its tutor qualities and usefulness than a non-adaptive robotic tutor.
• H5: The learning experience with an adaptive robotic tutor is rated better than with a non-adaptive robotic tutor.

## 2 Materials and Methods

### 2.1 Robot-Supported Learning Environment

We implemented a stand-alone robot-supported learning environment as a complementary addition to an existing course, in the form of an individual tutoring for exam preparation. This tutoring consisted of three learning sessions taking place within 10 weeks.

The Pepper robot^1^ was chosen as an interactive tutor because its integrated tablet offers the opportunity to visually present the learning material and is a consistent way to interact with the robot, because voice recognition is still prone to errors. In addition to the visual presentation of the learning material on the tablet, it is also presented vocally by the robot. The learner interacts with the learning environment *via* touch input on the tablet and the robot responds in a multi-modal manner using different communicative channels such as speech and gesture, scaffolding the social component of learning.

**FIGURE 1:**
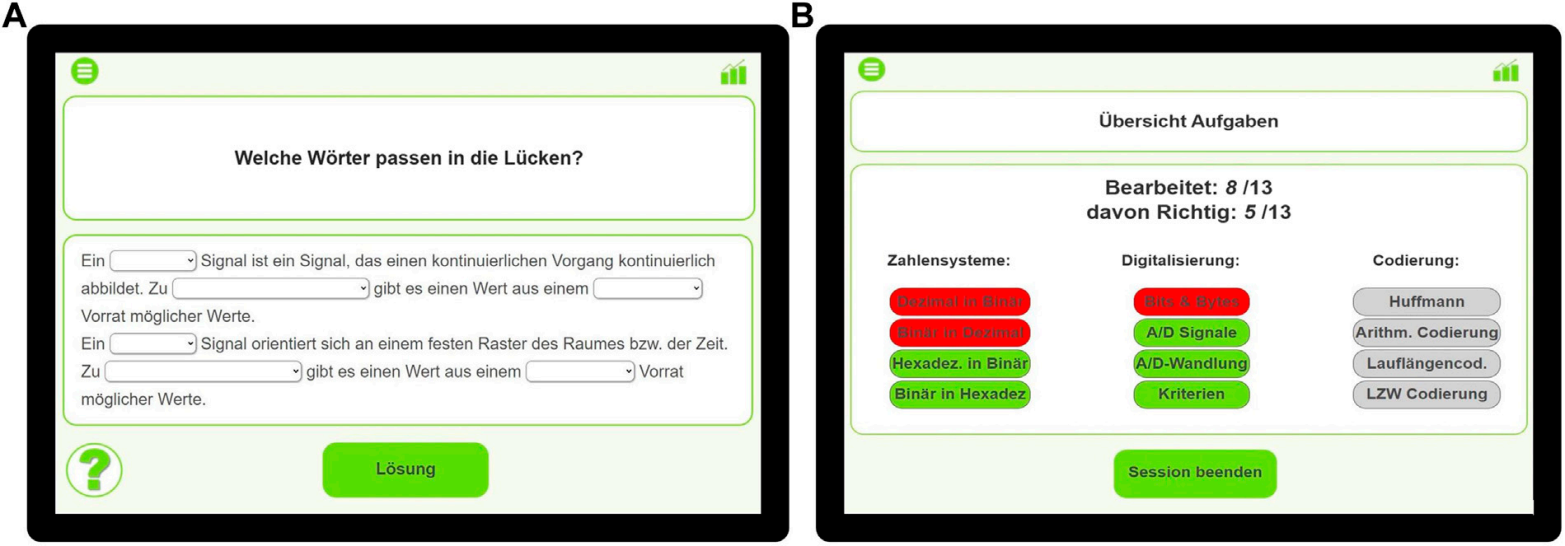
Exemplary pages of the learning environment. **Left:** example exercise. **Right:** exercise overview.

The learning environment starts with a short introduction by the robot. Because there is no predefined structure, the learner is able to select the topics he or she wants to practice in a free sequence. After the learner chooses a topic, the related exercise is displayed on the screen and verbalized by the robot. The learner is then able to choose an answer option in the tablet. Depending on the user’s answer, the robot gives adequate feedback: In case of a correct answer, the robot praises the learner; in case of an incorrect answer, the robot encourages the participant and gives explanations on the correct solution. There is also an opportunity to repeat the solution, and most of the exercises offer a help button to receive a hint on the solution by the robot if needed. In addition, there is an overview page with all exercises divided into subtopics and displayed in different colors depending on whether they are undone (gray), done correctly (green), or done incorrectly (red). From this overview page, the learner can directly navigate to each exercise, allowing to easily repeat incorrect exercises. As time to process the learning environment is limited, we implemented a timer, so the robot reminds the users of time limit 10 min before the session is over and kindly asks them to end the learning session when time is over. An exemplary exercise and the overview page are shown in Figure 1.

#### 2.1.1 Learning Material

The learning materials contain exercises of all topics of the undergraduate course “Digital Media” at the University of Wuerzburg, Germany. The first learning session includes the topics number systems, digitalization, and coding algorithms; the second learning session includes the topics audio and text; and the third learning session contains the topic images and all exercises of the previous sessions. Each of the topics is divided into three to four subtopics, which contain between one and five exercises. This structure is supposed to simplify navigation and finding the exact topic the learner wants to practice. In total, the three learning sessions contain 48 exercises. Because the robot-supported tutoring is meant to be a repetition and additional practice of the courses’ content, there were no prior learning units. There are different types of exercises such as single choice (23), multiple choice (8), cloze texts (10), and assignment exercises (7).

#### 2.1.2 Implementation of the Adaptive Robotic Tutor

We implemented two versions of the robotic tutor: adaptive and non-adaptive. In both conditions, we set up the learning environment to respond to the learner’s input using the robot’s speech and nonverbal behaviors and visual cues on the robot’s tablet, such as highlighting the correct answer options. The non-adaptive condition is limited to the functionality described above. In the adaptive condition, adaptation of the robot’s behavior was realized in several ways. There are adaptations that occur in each session, and adaptations over time that occur in the second and third learning sessions that refer to the previous session(s).

At the beginning of each learning session, the robot asks the learner to sign in with their individual participation code, which is then connected to the participant’s performance data in the learning environment. In the first session, the robot additionally asks participants for their name and uses the name to address the learner with his or her name in this and the following sessions (e.g., “That was correct, well done Laura”). Furthermore, the robot adapts its feedback based on the user’s answer: if the answer is correct, this is briefly noted. If the answer is incorrect, the robot first explains which option is correct and additionally explains why the user’s answer is wrong, depending on the chosen incorrect answer and thus gives hints on how to avoid such mistakes in the future (e.g., adaptive answer “Unfortunately this is not correct. The signal’s required storage space is 21 bytes. The *y*-axis shows that the signal is quantized with 8 bits, because the highest value from the scale 132 is quantized with 8 bits. Then we count the red sampling points, which are twenty-one. So you calculate 21 times 8 bits and come to 21 bytes. Maybe your result was 19 bytes because you have quantized with 7 bits or have miscounted with the sample points?”). Additionally, the robot tries to detect whether the user is struggling with an exercise, and to support by offering help. The robot only offers to help in case the user takes significantly longer for the exercise than usual. Thus, we implemented a timer to each exercise with the estimated processing time of each exercise. In case there is no input of the user on the tablet until the timer is over, the robot offers to give a hint on the exercise if the user clicks the help button (e.g., “Do you want me to give you a hint? Then click the help button”).

After completing a subtopic, depending on the user’s performance in the according exercises, the robot summarizes the performance, praises the learner (e.g., “Well done! You already know a lot about color models. Keep it up Laura!”) if more than 50% of the exercises were correct, or encourages the learner in case of a poor performance and gives advice on how to improve in that topic. (e.g., “That didn’t go well, Laura. Nevertheless, don’t get discouraged! If you practice a bit more, I’m sure you’ll know everything soon. The best way to learn coding is by practicing tasks like the one we did together”).

The robot gives personalized advice depending on the user’s performance when visiting the overview page. In the first and second learning sessions, the robot suggests to repeat a subtopic if more than 50% of the exercises of the subtopic were incorrect (e.g., “Things didn’t go that well with the topic of digitalization. Better have a second look at the wrong exercises”). In the last learning session, the user’s data from the first two sessions are also used and the exercise in the overview page are colored according to the performance from the first two learning sessions. For example, an exercise that was completed correctly in the first learning session is colored green in the third learning session, while a task that was completed incorrectly in the second session is now colored red. This gives the user an immediate overview of the performance of the previous learning sessions. The robot points this out to the user and suggests repeating the wrong tasks from the previous sessions (e.g., “Look Laura, here you can see your performance of the first two learning sessions. Now you can practice precisely the tasks you got wrong the first time”). Because data from the first two learning sessions are needed, this adaptation only occurs in session three.

Finally, when the user is asked by the robot to end the learning session by pressing a button, the robot gives recommendations on what to repeat and practice at home, based on the performance: if more than 50% of a subtopic were wrong, the robot suggests to particularly repeat and practice that topic at home (e.g., “UTF-8 encoding and decoding has not been done correctly yet. I recommend you to practice this topic in particular. The best way to learn it is to work on more practical exercises”). If the participants got more than 50% right in every subtopic, the robot praises the user for his or her good performance and encourages the user to keep going.

In the non-adaptive control condition, the robot’s behavior is the same for every participant. The robot does not call the participants by their names, gives the same explanation on the exercise independent from the chosen answer option, does not offer help (however, the help button is there and can be clicked), and gives no individual recommendations on what to repeat and practice at home. There is also no summary of the performance of the last sessions in the exercise overview page.

### 2.2 User Study

The user study contained three learning sessions at different points in time of the semester. It was accompanying the course “Digital Media 1” of the University of Wuerzburg in the winter semester 2020/2021. The time between two sessions was 2 to 4 weeks. The duration of each session varied between 35 and 45 min, depending on the number and kind of exercises. For the study, we compared two experimental conditions (control, adaptive) as between factor across all three sessions (sessions 1–3) as within factor in a mixed design. This study was approved by the local ethics committee of the Institute Human-Computer Media of the Julius-Maximilians University Würzburg. The possibility to take part in the study was offered to all students of the course and participation was voluntary at any point. There were no disadvantages for students who did not participate in the study. Participants received partial course credit for taking part in a study independently of the course. After the exam for the course, a short voluntary post-experimental survey was conducted, independent of course credit. Self-reported grades from participants who also took part in the post-experimental survey were compared to the average performance of the course.

#### 2.2.1 Participants

A total of *N* = 60 first-year undergraduate students took part in the study. Two participants had to be excluded because of technical errors during the interaction, resulting in *N* = 58 (52 female participants, 6 male participants) for the final sample with a mean age of *M* = 19.72 (SD = 1.45) and with all participants stating that they intended to write the exam at the end of the semester. Based on balanced allocation, 28 participants were assigned to the control condition and 30 were assigned to the adaptive condition. The higher percentage of female participants is due to the typical gender distribution of the study program, in which women are predominantly enrolled. Most of the participants (*n* = 43) had no prior experience with the robot before the first study of our series while some saw it on TV or the internet and only one participant stated to have met the robot before but without interacting. For the voluntary post-experimental questionnaire, 21 participants of the control condition and 22 participants of the adaptive condition decided to take part.

#### 2.2.2 Measurements

As a manipulation check, we asked how far participants agree to certain statements concerning adaptivity, e.g., “The robot has adapted its behavior to me”. To measure participants’ subjective knowledge gain, we asked them to rate their knowledge about each topic before and after processing the learning environment on a scale from “1—very bad” to “7—very good”. Situational motivation in reaction to the tutoring was measured using the Situational Motivation Scale (SIMS) (Guay et al., 2000), which consists of 16 items rated from “1—corresponds not at all” to “7—corresponds exactly”. To assess the participants’ intrinsic motivation concerning the content of the course, we used the subscale Interest/Enjoyment of the Intrinsic Motivation Inventory (IMI) (Ryan et al., 1983) and measured it before and after processing the learning environment. It contains 9 items on a scale from “1—not at all true” to “5—very true”. The evaluation of the learning environment and the robot’s capacities as a tutor were measured using the subscales Tutor Quality and Perceived Usefulness of the E-Learning Acceptance Measure (ElAM) (Teo, 2010) with 17 items rated on a scale from “1—totally disagree” to “7—totally agree”. Additionally, we specifically asked participants whether they had fun or were frustrated during the tutoring, if they would recommend it to other students, and whether they liked the robot using the same scale as the ElAM. In the last study of the series, after working with the robot for a while and getting the maximum of adaptation in the adaptive condition, we additionally surveyed the satisfaction of the three psychological needs using the subscales Autonomy, Relatedness, and Competence using the scale by Sheldon (Sheldon and Filak, 2008). The subscales contained three items, each ranging from “1—strongly disagree” to “5—strongly agree”.

At the end of each questionnaire, there was an opportunity to provide voluntary free text comments on the learning unit.

Approximately 6 weeks after the last session when participants had received their exam grades, all participants were asked to anonymously provide their grades in order to find potential differences in the exam performance. Additionally, we repeated the items of the manipulation check and the items on perceived fun, frustration, recommendation, and likability of the robot. Finally, we asked how far they perceived the learning environment retrospectively as useful for the exam and if they would use this offer again in the upcoming semester.

Each participant created an individual code (e.g., containing certain letters of their parents’ names) that is entered at the beginning of each session and the questionnaires to guarantee linked but anonymous data collection. We were able to correctly assign all data sets to the corresponding codes.

#### 2.2.3 Procedure

**FIGURE 2:**
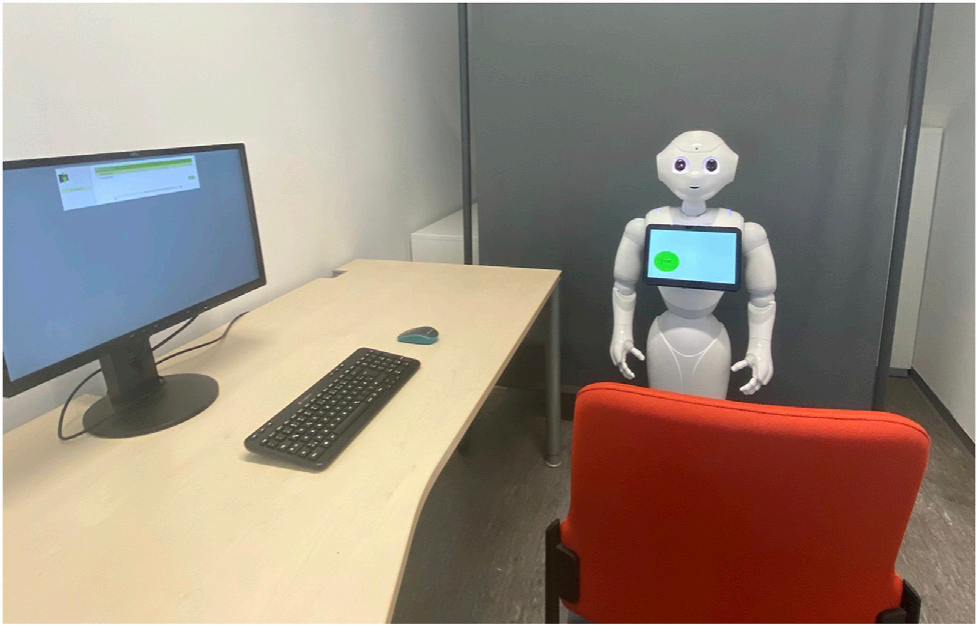
Experimental setup.

The procedure for all three parts of the study was identical and took place in the same lab room of the University of Wuerzburg. One participant attended a session at a time. Each participant was welcomed by the experimenter, was asked to read and sign a consent form, and was seated on a chair next to a computer and in front of the robot. First, the experimenter asked the participants to fill out a short questionnaire (3 min) at the computer and then to start the learning session by clicking on the robot’s tablet. On the table next to the participant, pen and paper were supplied, which were needed for some exercises. The time to spend in the learning environment was limited to 35–45 min (depending on the session). The robot gave a hint 10 min before time was over and asked the participant to end the learning session after time ran out. Before saying goodbye, the robot asked the participant to complete the questionnaires (10–15 min) at the computer afterwards. To minimize disturbance, the experimenter waited behind a partition wall during the experiment. The experimental setup can be seen in Figure 2.

## 3 Results

Due to the scope of the study, we differentiate the effect of three factors. For all analyses, we include the factor condition (control, adaptive) to assess the influence of the experimental manipulation. The within-factor session (sessions one to three) addresses changes across the three tutoring sessions. Finally, the within-factor pre/post (pre and post tutoring) is used to investigate the change from prior to a tutoring session to after the session, requiring two measures within one session. This pre/post factor was only investigated for two measures, namely, subjective knowledge and IMI score. The only analysis taking all three factors into account was the change in IMI scores, which were measured twice in each respective session. This analysis was not conducted for the subjective knowledge, due to different content across the three sessions. Analyses for the post-exam survey only differentiate between both conditions.

We conducted a 2 × 3 mixed ANOVA with the between-factor condition (control, adaptive) and the within-factor session (sessions one to three) on the mean of the three items for the manipulation check (see Tables 3, 4). We found a significant effect of the condition, with higher values in the adaptive condition relative to the control condition for all sessions. There was also a significant effect of session, with higher values for the first session relative to both subsequent sessions. The interaction was not significant.

The retrospective check for the manipulation in the post-exam survey with a reduced number of participants was significant *t* (41) = 3.38, *p* = 0.002 with higher values in the adaptive condition (*n* = 22, *M* = 5.17, SD = 1.00) compared to the control condition (*n* = 21, *M* = 3.92, SD = 1.39).

### 3.1 Learning Performance

For H1, we conducted an ANOVA for the end of semester exam and compared the adaptive and control condition and the average course performance (excluding the grades of study’s participants), resulting in three conditions. There was a significant effect of condition *F* (2, 162) = 43.16, *p* = 0.020, $\eta_{p}^{2}$ = 0.20. Bonferroni-corrected post-hoc tests showed that participants in the adaptive condition (*n* = 22, *M* = 1.85, SD = 0.49) performed significantly better relative to the average course performance (*n* = 122, *M* = 2.79, SD = 1.21; *p* = 0.002). Participants in the control condition (*n* = 21, *M* = 2.03, SD = 0.84) also performed significantly better relative to the average of the course (*p* = 0.019), while both experimental conditions did not differ significantly (*p* = 1).

### 3.2 Subjective Knowledge

To address H2, we conducted separate 2 × 2 mixed ANOVAs with the between-factor condition (control, adaptive) and the within-factor pre/post (pre and post tutoring) for every session, using the mean of the participants’ self-assessed knowledge in all topics within the respective session (see Table 2). All descriptive values are shown in Table 1. For the first session (number systems, digitalization, and coding algorithms) there was a significant main effect of pre/post with higher values post tutoring. The main effect for condition was not significant, and there was no significant interaction.

**TABLE 1:** Mean values for subjective knowledge of all subjects within each session on a scale of 1–7. Standard deviations in parentheses.

	Session 1		Session 2		Session 3	
	Pre	Post	Pre	Post	Pre	Post
Control	3.78 (0.73)	5.29 (0.82)	3.61 (0.85)	3.68 (1.06)	4.38 (0.89)	5.08 (0.89)
Adaptive	3.83 (0.65)	5.04 (0.70)	3.67 (0.86)	3.65 (1.28)	4.27 (0.83)	5.11 (0.62)

**TABLE 2:** Results of 2 × 2 mixed ANOVAs for subjective knowledge. * indicates significance.

Session	Condition (control, adaptive)–*F* (1, 56)	Pre/post (pre and post tutoring)–*F* (1, 56)	Interaction–*F* (1, 56)
Session 1	*F* = 0.38, *p* = 0.538, $\eta_{p}^{2}$ = 0.01	*F* = 154.18, *p* < 0.001, $\eta_{p}^{2}$ = .73*	*F* = 1.75, *p* = 0.191, $\eta_{p}^{2}$ = 0.03
Session 2	*F* = 0.00, *p* = 0.949, $\eta_{p}^{2}$ = 0.00	*F* = 0.05, *p* = 0.831, $\eta_{p}^{2}$ = 0.00	*F* = 0.12, *p* = 0.732, $\eta_{p}^{2}$ = 0.00
Session 3	*F* = 0.05, *p* = 0.828, $\eta_{p}^{2}$ = 0.00	*F* = 52.32, *p* < 0.001, $\eta_{p}^{2}$ = 0.48*	*F* = 0.41, *p* = 0.524, $\eta_{p}^{2}$ = 0.01

In the second session (audio and text), there were no significant main effects of pre/post and of condition, and there was no interaction.

Finally, for the third session (images and repetition of the content of both previous sessions), there was a significant main effect of pre/post with higher values post tutoring. The main effect for condition was not significant and there was no significant interaction.

### 3.3 Motivation

**FIGURE 3:**
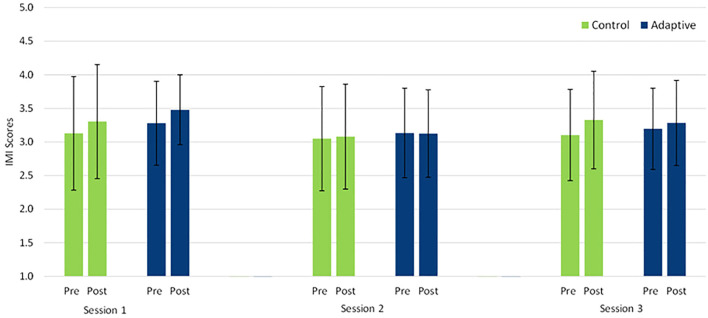
IMI values prior to and post tutoring for both conditions across all sessions. Error bars represent SDs.

To investigate H3, we conducted a 2 × 2 × 3 mixed ANOVA with the between-factor condition (control, adaptive), the within-factor session (session one to three), and the within-factor pre/post (pre and post tutoring) on the IMI score (see Figure 3). There was no significant effect of condition *F* (1, 56) = 0.26, *p* = 0.609, $\eta_{p}^{2}$ = 0.01, but there were a significant effect of session *F* (2, 112) = 6.74, *p* = 0.002, $\eta_{p}^{2}$ = 0.11 and of pre/post *F* (1, 112) = 10.91, *p* = 0.002, $\eta_{p}^{2}$ = 0.16. The two-way interactions of condition with session (*p* = 0.446) and with pre/post (*p* = 0.486) were not significant; however, the interaction between session and pre/post was significant *F* (2, 112) = 3.45, *p* = 0.035, $\eta_{p}^{2}$ = 0.06. The three-way interaction was not significant (*p* = 0.523).

In addition, we conducted three 2 × 3 mixed ANOVAs (see Table 4) with the between-factor condition (control, adaptive) and the within-factor session (sessions one to three) for the intrinsic, identified, and extrinsic scale of the SIMS (see Table 3). For situational intrinsic motivation, we found no significant effect of condition, but a significant effect of session. There was no significant interaction. Bonferroni-corrected post-hoc tests showed significantly higher values in session one relative to session two (*p* < 0.001) as well as session three (*p* = 0.012) as well as a significantly higher value in session three relative to session two (*p* < 0.001).

**TABLE 3:** Mean values for questionnaire measures across both conditions each on a scale of 1–7. Standard deviations in parentheses.

		Session 1	Session 2	Session 3
Manipulation check	Control	4.88 (1.34)	4.13 (1.36)	4.14 (1.53)
	Adaptive	5.99 (0.75)	5.48 (0.84)	5.49 (1.00)
SIMS intrinsic	Control	5.99 (0.88)	5.25 (1.14)	5.66 (1.20)
	Adaptive	6.13 (0.82)	5.44 (1.13)	5.83 (1.03)
SIMS identified	Control	6.37 (0.76)	6.40 (0.62)	6.54 (0.54)
	Adaptive	6.53 (0.46)	6.40 (0.70)	6.58 (0.54)
SIMS extrinsic	Control	5.48 (0.44)	5.20 (0.58)	5.30 (0.55)
	Adaptive	5.37 (0.62)	5.17 (0.52)	5.17 (0.68)
Tutor quality	Control	6.01 (0.77)	5.77 (0.80)	5.63 (1.04)
	Adaptive	6.05 (0.61)	5.60 (0.87)	5.70 (0.88)
Perceived usefulness	Control	5.80 (0.72)	5.38 (0.94)	5.78 (0.76)
	Adaptive	5.76 (0.66)	5.30 (0.85)	5.59 (0.80)
Perceived fun	Control	6.46 (0.84)	5.54 (1.23)	5.96 (1.20)
	Adaptive	6.57 (0.77)	5.50 (1.17)	6.00 (0.98)
Perceived frustration	Control	1.57 (0.96)	3.18 (1.76)	2.46 (1.67)
	Adaptive	2.13 (1.11)	3.77 (1.68)	3.13 (1.48)
Sympathy for robot	Control	6.21 (1.17)	5.79 (1.69)	5.64 (1.62)
	Adaptive	5.97 (1.45)	5.70 (1.24)	5.53 (1.61)
Recommendation	Control	6.32 (0.77)	6.21 (0.96)	6.39 (1.07)
	Adaptive	6.50 (0.90)	6.27 (0.98)	6.27 (0.98)

For situational identified motivation, we found no significant effect of condition and no significant effect of session. The interaction was also not significant.

For situational extrinsic motivation, we found no significant effect of condition, but a significant effect of session. There was no significant interaction. Bonferroni-corrected post-hoc tests showed significantly higher values in session one relative to session two (*p* = 0.014) but no significant difference to session three (*p* = 0.069). There was also no significant difference between session two and session three (*p* = 1).

### 3.4 Tutor Quality and Perceived Usefulness

To address H4, we conducted two 2 × 3 mixed ANOVAs (see Table 4) with the between-factor condition (control, adaptive) and the within-factor session (sessions one to three) on the perceived tutor quality and the perceived usefulness (see Table 3). For the tutor quality, there was no significant effect of condition but a significant effect of session. The interaction was not significant. Bonferroni-corrected post-hoc tests showed significantly higher values in session one relative to session two (*p* = 0.004) and to session three (*p* = 0.002) but no significant difference between sessions two and three (*p* = 1).

**TABLE 4:** Results of 2 × 3 mixed ANOVAs for selected measures. * indicates significance.

Measure	Condition (control, adaptive)–*F* (1, 56)	Session (sessions one to three)–*F* (2, 112)	Interaction–*F* (2, 112)
Manipulation check	*F* = 22.72, *p* < 0.001, $\eta_{p}^{2}$ = 0.29*	*F* = 15.17, *p* < 0.001, $\eta_{p}^{2}$ = 0.21*	*F* = 0.55, *p* = 0.577, $\eta_{p}^{2}$ = 0.01
SIMS intrinsic	*F* = 0.48, *p* = 0.491, $\eta_{p}^{2}$ = 0.01	*F* = 21.60, *p* < 0.001, $\eta_{p}^{2}$ = 0.28*	*F* = 0.26, *p* = 0.967, $\eta_{p}^{2}$ = 0.00
SIMS identified	*F* = 0.02, *p* = 0.904, $\eta_{p}^{2}$ = 0.00	*F* = 2.43, *p* = 0.093, $\eta_{p}^{2}$ = 0.04	*F* = 0.62, *p* = 0.542, $\eta_{p}^{2}$ = 0.01
SIMS extrinsic	*F* = 0.63, *p* = 0.431, $\eta_{p}^{2}$ = 0.01	*F* = 5.17, *p* = 0.007, $\eta_{p}^{2}$ = 0.08*	*F* = 0.26, *p* = 0.775, $\eta_{p}^{2}$ = 0.01
Tutor quality	*F* = 0.02, *p* = 0.904, $\eta_{p}^{2}$ = 0.00	*F* = 9.14, *p* < 0.001, $\eta_{p}^{2}$ = 0.14*	*F* = 0.82, *p* = 0.443, $\eta_{p}^{2}$ = 0.01
Perceived usefulness	*F* = 0.36, *p* = 0.550, $\eta_{p}^{2}$ = 0.01	*F* = 9.63, *p* < 0.001, $\eta_{p}^{2}$ = 0.15*	*F* = 0.29, *p* = 0.747, $\eta_{p}^{2}$ = 0.01
Perceived fun	*F* = 0.02, *p* = 0.886, $\eta_{p}^{2}$ = 0.00	*F* = 34.54, *p* < 0.001, $\eta_{p}^{2}$ = 0.38*	*F* = 0.17, *p* = 0.848, $\eta_{p}^{2}$ = 0.00
Perceived frustration	*F* = 4.16, *p* = 0.046, $\eta_{p}^{2}$ = 0.07*	*F* = 28.85, *p* < 0.001, $\eta_{p}^{2}$ = 0.34*	*F* = 0.03, *p* = 0.967, $\eta_{p}^{2}$ = 0.00
Sympathy for the robot	*F* = 0.19, *p* = 0.666, $\eta_{p}^{2}$ = 0.00	*F* = 5.23, *p* = 0.007, $\eta_{p}^{2}$ = 0.09*	*F* = 0.15, *p* = 0.860, $\eta_{p}^{2}$ = 0.00
Recommendation	*F* = 0.02, *p* = 0.875, $\eta_{p}^{2}$ = 0.00	*F* = 1.51, *p* = 0.225, $\eta_{p}^{2}$ = 0.03	*F* = 1.22, *p* = 0.299, $\eta_{p}^{2}$ = 0.02

For the perceived usefulness, there was no significant effect of condition but a significant effect of session. The interaction was not significant. Bonferroni-corrected post-hoc tests showed significantly lower values in session two relative to session one (*p* = 0.001) and to session three (*p* = 0.002) but no significant difference between sessions one and three (*p* = 1).

### 3.5 Learning Experience

To investigate H5, we conducted four 2 × 3 mixed ANOVAs (see Table 4) with the between-factor condition (control, adaptive) and the within-factor session (sessions one to three) on the perceived fun, perceived frustration, sympathy for the robot, and likelihood to recommend the tutoring to fellow students (see Table 3). For perceived fun, we found no significant effect of condition, but a significant effect of session. There was no significant interaction. Bonferroni-corrected post-hoc tests showed significantly higher values in session one relative to both other sessions (*p* < 0.001) and a significantly higher value in session three relative to session two (*p* = 0.001).

For perceived frustration, we found a significant effect of condition with higher frustration values for the adaptive condition. There was also a significant effect of session. The interaction was not significant. Bonferroni-corrected post-hoc tests showed significantly higher values in session one relative to both other sessions (*p* < 0.001) and a significantly higher value in session three relative to session two (*p* = 0.001).

For sympathy for the robot, we found no significant effect of condition, but a significant effect of session. There was no significant interaction. Bonferroni-corrected post-hoc tests showed significantly higher values in session one relative to session three (*p* = 0.014) but no significant difference to session two (*p* = 0.095). There was also no significant difference between sessions two and three (*p* = 0.906).

For recommendation, we found no significant effect of condition and no significant effect of session. The interaction was also not significant.

In the post exam survey with a reduced number of participants, we found no significant effect of the condition on perceived fun, *t* (41) = 0.47, *p* = 0.644, sympathy for the robot, *t* (41) = 0.21, *p* = 0.838, or for recommendation, *t* (41) = 0.86, *p* = 0.395. There was a significant difference in perceived frustration, *t* (41) = 2.24, *p* = 0.031, with lower values in the control condition (*n* = 21, *M* = 2.14, SD = 1.31) relative to the adaptive condition (*n* = 22, *M* = 3.05, SD = 1.33).

### 3.6 Free Text Comments

Across all sessions, the opportunity to provide written feedback at the end of each questionnaire was used 74 times, which is 42.53% of the cases. For a systematic evaluation, we regarded each single comment independent of session and participant but connected to one of the two conditions. First, we sorted the comments into negative, positive, and neutral (neither positive nor negative). We then derived categories from the most frequently mentioned statements, resulting in the categories below. We only reported comments with a minimal count of five.

The systematic evaluation revealed that the most frequent comment referred to praise for the tutoring in general such as the possibility for participation and the idea (*n* = 16, e.g., “Thank you for the opportunity to learn with Pepper”, “Nice and very interesting idea!”). It was also highlighted that the tutoring was helpful (*n* = 5) and there was specific praise for the robot (*n* = 5, e.g., “Pepper explains the tasks in a pleasant and understandable way.”). On the other hand, some comments pointed out technical difficulties and bugs (*n* = 10). Another nine times, it was commented that the robot talks too much, seven of them in the adaptive condition. The hand movements and gestures of the robot were described as annoying in 8 cases. Also, the robot was partly described as impatient (*n* = 5); this occurred exclusively in the adaptive condition.

## 4 Discussion

In this paper, we presented a field study with three learning sessions with our robotic tutor in order to further investigate the general applicability of social robots as tutors in higher education, and in particular, to assess if adaptivity of the tutor can additionally benefit learning compared to a non-adaptive version of the robot.

First, the participants perceived the robot’s adaptive behavior as the manipulation check for the adaptivity manipulation was significant. Taking part in the robotic tutoring resulted in significantly better exam grades; however, the experimental conditions did not differ significantly, only partially supporting H1. The subjective knowledge gain during the tutoring was significant for all topics in session one, none in session two, and all but one in session three. We found no significant effect of condition or an interaction, indicating a benefit for the tutoring overall, but not specifically for the adaptive version, therefore rejecting H2. In regard to motivation, there were no main effects of condition for intrinsic motivation as measured by the IMI, as well as all subscales of situational motivation, therefore rejecting H3. However, the significant benefit of the tutoring in general on the post scores for the IMI and the significant effects of session have to be considered in more detail in Subsection 4.1 and Subsection 4.2. The rating of the tutor qualities and the perceived usefulness of the robotic tutor did not differ significantly between conditions. Therefore, we have to reject H4, similarly to the previous measures, and the significant differences across sessions will be discussed in Subsection 4.2. For the rating of the learning experience based on the perceived fun, sympathy for the robot, and likelihood to recommend the tutoring to fellow students, there were no significant effect of condition. Concerning perceived frustration, we found a significant effect, but with higher frustration in the adaptive condition. Thus, we reject H5 and discuss the significant differences across sessions in Subsection 4.3.

### 4.1 Main Results

To summarize the main findings, we found evidence that, independently of the adaptation, the robotic tutoring provides objective and subjective benefits for the participants. Participants taking part in the tutoring showed significantly better exam performance compared to the average performance of the course. Albeit there were no differences between the two conditions, it demonstrates that opting for a session with the robotic tutor is beneficial. However, these results need to be considered carefully as self-selection in participation might have influenced these results and students who participate voluntarily in the additional tutoring might have a higher motivation in general. Nonetheless, the comparison of exam grades with the non-participants is of high practical relevance and the group of students who prefer self-study and students who prefer studying together with the robot might be comparable.

This is also supported by the participants’ significant subjective knowledge gain after each session of the robotic tutoring for all topics in the first and most of the third learning session.

Additionally, we found significant but less straightforward effects on participants’ motivation, with no significant effect of the manipulation. Results of the IMI showed a positive effect of the tutoring on the intrinsic motivation to deal with the topics of the course, with generally higher values after the sessions relative to prior. This suggests that such a robotic tutoring session might have a positive short-term effect on motivation regarding this course. There is no clear pattern of IMI scores gradually increasing across sessions, indicating no additive effect of intrinsic motivation increase, which is supported by an accompanying pattern of results for situational motivation. However, the change of motivation appears to be more complex in regard to its change across the semester and is discussed conjointly with other effects of session in the subsequent section.

Ratings for the robot, the learning environment, and the learning experience were generally high, such as tutor quality, perceived fun, and likability of the robot with mean values above 5.5 (scale from 1 to 7). In particular, the item “I would recommend the tutoring to fellow students” was rated high with mean values above six in all of the learning sessions. These positive results are supported by the fact that there was a dropout rate of zero percent over the three sessions.

These benefits strongly support the robotic tutoring as a whole but appears to be independent of the adaptivity manipulation.

### 4.2 Time-Based Effects

There were significant changes over time on almost all scales, including motivation and the perception of the robot. It is especially noticeable that most of the scales had the highest ratings in the first session (e.g., subscale intrinsic motivation of the SIMS, fun, tutor quality, and likability of the robot) and the lowest ratings in the second session (e.g., perceived usefulness, fun, and subscale intrinsic motivation of the SIMS). The third session was mostly rated moderately.

We attribute this trend to two effects, especially because this pattern was identical across both conditions: first, the novelty effect of the tutoring with the social robot, and second, the overall motivation of students across the semester. Only some participants stated that they had prior experience with the robot, it is likely to be the first time they interacted with the Pepper robot for a longer period of time. We consider this to be a likely explanation, because especially the scales directly related to the robot such as likability, tutor quality, and fun were rated significantly higher in the first learning session.

The significantly lower ratings across most of the scales for the second session might also be influenced by the time of the session within the semester. Because nearly all indicators were systematically low in session two for a learning environment, which was identical with the exception of the learning content to the previous and subsequent session, we suspect an external cause.

After a probable high motivation of the students at the beginning of the semester (first session), the overall motivation in the middle of the semester decreases (second session) before students have to get back to a higher motivational level in the time before exams start (third session). This assumption is further supported by the fact that the scores for motivation measured by the IMI before processing the session were significantly lower in session two than in sessions one and three. Finally, even though there was no subjective knowledge gain in the topics audio and texts in the second session, the subjective knowledge gain for both topics was significant for the third session, even though the exercises were identical.

### 4.3 Group-Based Effects

We found that participants in the non-adaptive condition were significantly less frustrated than those in the adaptive condition. First, frustration levels were low in general, with an average of 2.70 on a seven-point scale. Because some participants stated dislike for long monologues of the robot, the additional feedback and recommendations by the robot in the adaptive condition might have resulted in this detrimental effect. An additional function to skip the robot’s speech may address this problem. However, the frustration might also be caused by the explanation of their mistakes to the learner during the exercises, which might lead to a negative attitude towards their own performance, resulting in more frustration.

There were no further significant differences between the experimental conditions. These findings are contrary to our expectation and the results of related work (Leyzberg et al., 2014; Leyzberg et al., 2018; Schodde et al., 2017). However, previous studies have focused mainly on children. As Belpaeme and Tanaka (2021) suggested, adults have higher expectations on the technical implementation of robots; thus, simple adaptations such as calling participants by their names might be less convincing. Because some participants noted in the free text comments that the robot talks too much, some kind of adaption such as the recommendations might have been perceived negatively.

Furthermore, the lack of significant differences between the two conditions might be a result of using a high-quality learning environment as a control group, which we consider ethically necessary because the tutoring is connected to an ongoing course. The learning material is equal in all conditions, and the robot always gives explanations on the solutions, so that the learning material itself creates a high personal relevance of the learning environment for the participants, which might influence intrinsic motivation. The fact that the ratings for the robot, the learning environment itself, and the learning experience were high in general supports this interpretation. We assume that this might prevent us from finding significant differences between the groups, which has also been a difficulty in previous studies.

On the other hand, the free text comments provide insights that perhaps not all adaptive elements are perceived as positive, such as the robot’s extended talking time when recommending tasks for repetition or the robot’s perceived impatience when offering help. Therefore, it might be possible that the positive effects of some adaptive elements are influenced by the negative perception of some other adaptive elements. This could result in the adaptive condition not being rated better in the overall evaluation. In the future, it will be important to find out which adaptive elements are really conducive to learning and which may actually worsen the learning experience.

### 4.4 Conclusion and Future Work

In this study, we presented a field study by integrating a robot-supported tutoring into an existing university course as an additional voluntary offer. In the tutoring, university students learned together with an adaptive or non-adaptive robot over three sessions during one semester. The aims of the study were to investigate to what extent a robotic tutor can be effective in higher education scenarios in general and particularly whether an adaptive robot can benefit the learning experience and outcomes.

The results demonstrate that opting in for a learning session with the robotic tutor is beneficial in terms of a significantly better exam grade, a subjective knowledge gain, and an increased intrinsic motivation to deal with the content of the course in general. However, the adaptation of the robot’s behavior seems to play a secondary role as there were no significant differences between the adaptive and non-adaptive conditions using our measurements for motivation and learning gains.

The main contribution of the research presented in this paper are an affirmation that social robot tutors can benefit the learning gains of students in higher education. While our study has not yielded the conclusions we predicted, it is nevertheless valuable in demonstrating the limitations and opportunities of using social robots in higher education. There is a clear need of more, and finer-grained, field studies in order to get deeper insights into possible application scenarios and benefits of robotic tutors at universities and therefore to address the challenges of supporting students in higher education.

In the future, we plan to continue our approach of integrating social robots into university teaching. Because we expect a motivational decline during the middle of the semester, we aim to counteract this by additional motivational features as well as trying to establish a baseline for comparison in non-participating students. Because there is no significant differentiation based on the adaptive behavior, we plan a different approach by allowing students to learn with both versions of the robotic tutor, allowing for a clear preference rating and allowing participants to indicate their preference for different adaptive mechanisms. In addition, we would like to address participants’ negative comments about the learning environment, particularly the ability to skip the robot’s speech, allow more time before the robot offers help, and minimize hand movements.

## Data Availability

The raw data supporting the conclusion of this article will be made available by the authors, without undue reservation.

## Footnotes

1 Pepper robot: https://www.softbankrobotics.com/emea/de/pepper.
